# Lateralized scale-eating behaviour of cichlid is acquired by learning to use the naturally stronger side

**DOI:** 10.1038/s41598-017-09342-7

**Published:** 2017-08-21

**Authors:** Yuichi Takeuchi, Yoichi Oda

**Affiliations:** 1 Department of Anatomy and Neuroscience, Graduate School of Medicine and Pharmaceutical Sciences, University of Toyama, Toyama, Japan; 2 Graduate School of Science, Nagoya University, Aichi, Japan

## Abstract

The scale-eating cichlid *Perissodus microlepis* exhibits significant lateralised predation behaviour using an asymmetric mouth. But how the acquisition of the behavioural laterality depends, if at all, on experience during development remains obscure. Here, naïve juveniles were tested in a series of predation sessions. Initially, they attacked both sides of the prey, but during subsequent sessions, attack direction gradually lateralised to the skewed mouth (dominant) side. Attack side preference of juveniles that had accumulated scale-eating experience during successive sessions was significantly higher than that of naïve juveniles at the same age and naïve adults. Thus, the lateralised behaviour was a learned experience, and did not develop with age. Surprisingly, however, both maximum amplitude and angular velocity of body flexion during attack of naïve fish was dominant on one side. Therefore, scale-eating fish have a naturally stronger side for attacking prey fish, and they learn to use the dominant side through experience.

## Introduction

Intraspecific variations in behaviour are a key factor in adaptability and fitness^1^. The preference for using one side of the body over the other, as observed typically in human handedness^2, 3^, is referred to as behavioural laterality. Even a basal lineage of vertebrates, hagfish, shows a lateral preference in coiling direction (clockwise/counter-clockwise coiling) at rest on an individual level^4^. Behavioural laterality has been demonstrated in every vertebrate class from fish to mammals and also in invertebrates^5^. Therefore, it is likely to have an ancient evolutionary origin^6^.

Lateralised behaviours are thought to be strengthened during development^7^. Little is known, however, about how they are acquired during development. Lateralisation is advantageous to foraging, defending against competitors, being vigilant against predators, or attending to prospective mates^8, 9^. Gombe chimpanzees, *Pan troglodytes*, that are more lateralised are more efficient when fishing for termites^10^. Similarly, Australian parrots with strong foot and eye preferences outperform less-lateralised individuals during demanding tasks^11^, and lateralised pigeons are also better in a visual discrimination task than their less-lateralised counterparts^12^. It has been suggested that lateral differences in human hand-use performance are acquired by learning and experience during growth^13^. A longitudinal study of infants showed that hand-use preference increases the strength of that preference over time^14^. By contrast, several genetic models have been proposed to explain that these asymmetries are directional^15–17^. To date, variation in handedness in human and other animals is likely attributable to the complex interactions between genetic and environmental factors^18, 19^. Development of brain asymmetry and visual lateralization was shown to be affected by light stimulation during the embryonic stage in chicks^20, 21^ and similarly in zebrafish^22^. These findings suggest that an interaction between genetic and environmental factors plays a key role in the establishment of behavioural laterality. However, we still do not know how behavioural laterality is acquired throughout an organism’s life. Here we have attempted to reveal how and when the behavioural laterality ontogenetically arises by using the scale-eating cichlid in Lake Tanganyika, *Perissodus microlepis*, at its developing stage.

**Figure 1:**
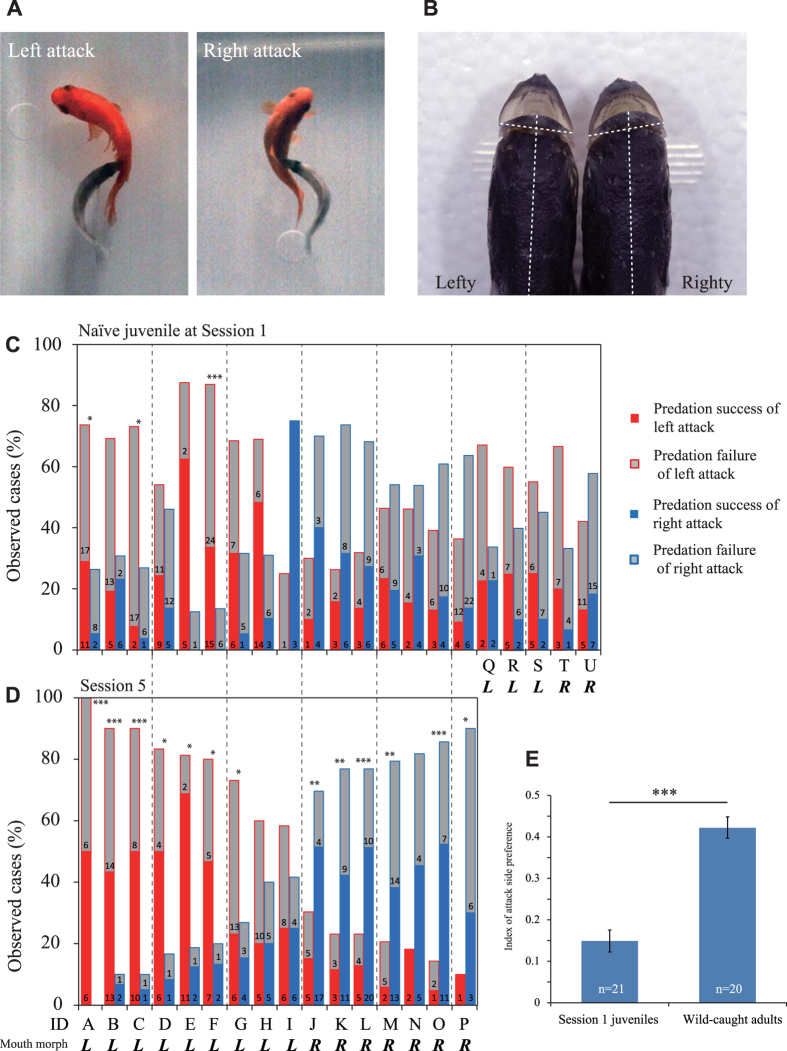
Attack side preference in *Perissodus microlepis*. (**A**) Photographs of left- and right-sided attacks. (**B**) Dorsal view of the mouth morphologies of lefty and righty fish. The dotted lines indicate the midline and the lateral tips of the lips. Change in the percentage of left-sided (red column) and right-sided (blue column) attacks in each juvenile predator from Session 1 (**C**) (N = 21 fish) to Session 5 (**D**) (*N*  = 16 fish). Grey columns indicate failed attempts at scale eating. The numbers at the bottoms of the columns indicate the number of attacks by each fish. Asymmetric mouth morphology, lefty (L) or righty (R), is denoted for each fish. P-values are from binomial tests. *P < 0.05; **P < 0.01; ***P < 0.001. (**E**) Weighted mean ± standard error index of attack side preference at Session 1 for juveniles and wild-caught adults. P-values are from the Wilcoxon rank-sum test. ***P < 0.001.

*P. microlepis* is an attractive model of behavioural laterality since the mouth is skewed either to the left or to the right, and adult fish exhibit conspicuously lateralised predatory behaviour (Fig. 1A and B) in that they nibble scales exclusively from one side of a prey fish’s body using the skewed mouth^23, 24^. Because the lefty and righty individuals coexist in the field population^24^, the mouth asymmetry is defined on an individual level but not on a population level^25^. Preferred attack orientation is concordant with the mouth opening direction, which involves skeletal asymmetry of the head and mouth^23^. This asymmetry is considered advantageous as it enlarges the contact area between the predator’s teeth and the prey’s trunk^26^. In fact, scale-eaters with more strongly skewed mouths eat more scales in the field^27^. The maximum angular velocity and amplitude of body flexion during a predatory attack, as observed in experiments with fish in tanks, is higher when a cichlid attacks on the dominant side of its mouth morphology^28^. The simplicity of laterality in *P. microlepis* should therefore facilitate understanding of the complex mechanisms of behavioural laterality.

It has been suggested that mouth asymmetry in scale-eaters is genetically determined^24, 29–31^, and a genome-wide association study showed that this trait has a genetic basis that is likely influenced by multiple loci^32^. Our previous study focused on the developmental process of behavioural laterality of predation in the scale-eater during large-scale fieldwork^27^. An individual’s preferred attack orientation was identified from the shapes of the foraged scales in its stomach. The analysis indicated that young juveniles (standard length [SL] < 45 mm) exhibit a weak bias for the attack side after scale-eating begins, and a preference for the attack side gradually strengthens as the fish grow. These results suggest that the remarkable behavioural laterality of adult scale-eaters is acquired after birth rather than being an innate behaviour.

In this study, we monitored, with high-speed cameras, the scale-eating behaviour of developing *P. microlepis* juveniles that were obtained from breeding in our laboratory, so as to examine the development of behavioural dynamics and the adaptive role of attack side preference. Through our behavioural experiment, we addressed three questions regarding behavioural laterality during predation: Do naïve juveniles with no scale-eating experience exhibit an attack side preference? How do individual attack side preference and behavioural kinetics change with successive predation experiments? Does enhancement of lateralised predation behaviour depend on internal factors associated with body growth or external factors such as scale-eating experience?

## Results

### Initial attack side preference of naïve fish

To examine behavioural laterality during individuals’ first experience of predation*, P. microlepis* juveniles (45.98 ± 0.77 mm SL, 21 fish, 4 months old) with no scale-eating experience were used to assess attack side preference in a tank. The naïve juveniles aggressively attacked prey goldfish that were introduced to the tank as prey. In the first predation experiment (Session 1), all juveniles attacked both sides of the prey fish (Supplementary Movie 1). Most of the naïve juveniles (18 out of 21 fish) attacked both sides subequally, whereas three individuals showed significant bias that favoured the skewed mouth direction (binomial test, *P* < 0.05; Fig. 1C). All naïve juveniles tested already had asymmetric mouths. The index of attack side preference (IAP: the rate of attacks from the dominant side corresponding to their asymmetric mouth) of the naïve juveniles was significantly lower, 0.149 ± 0.027 (weighted mean ± standard error [SE], *N* = 21), than that of wild-caught adult *P. microlepis* (Fig. 1E, IAP: 0.422 ± 0.025, mean ± SE; *N* = 20, Wilcoxon rank-sum test: z = 5.024, *P* < 0.001).

### Acquiring behavioural laterality through practice

**Figure 2:**
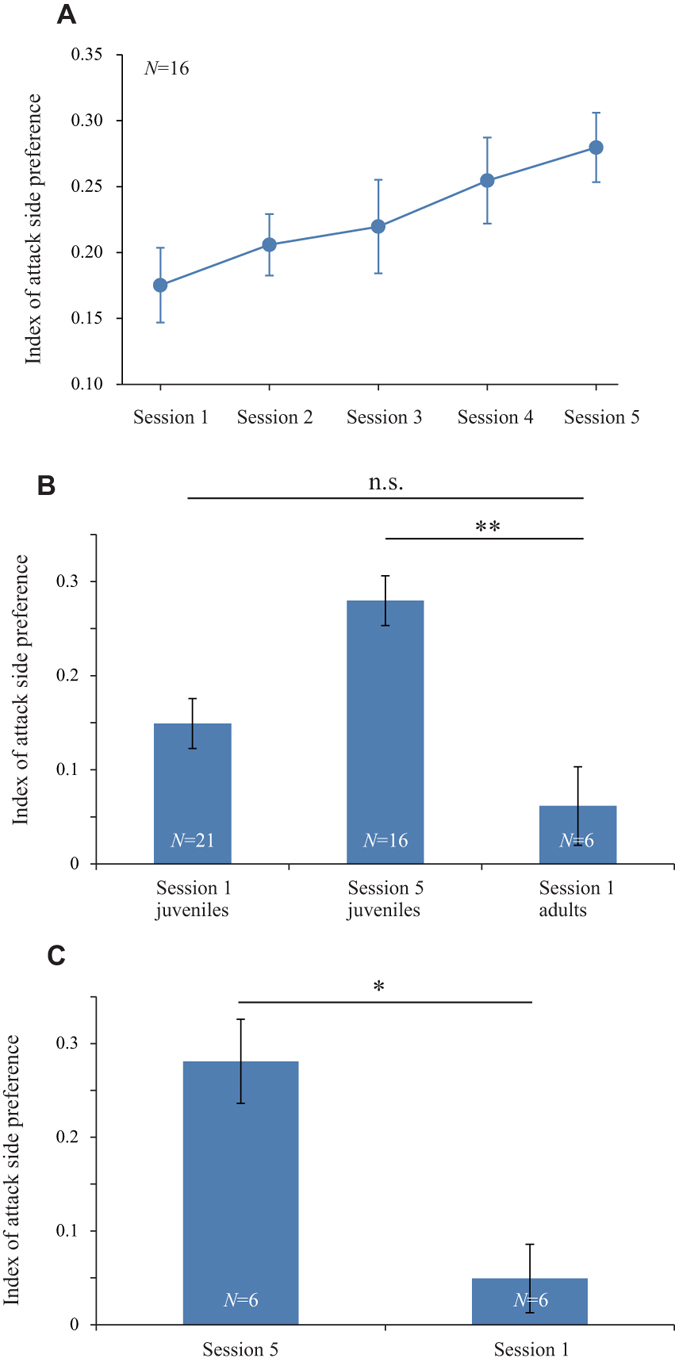
Comparison of attack side preference during repeated experiments. (**A**) The temporal change in attack side preference from Sessions 1 to 5 (mean ± standard error [SE], *N*  = 16 fish). (**B**) The attack side preference during Sessions 1 and 5 for juveniles and Session 1 for adults. P-values are from the Wilcoxon rank-sum test. (**C**) Differences in the level of attack side preference between Sessions 5 and 1 conducted on the same day of age. P-values are from the Wilcoxon signed-rank test. *P < 0.05. **P < 0.01. n.s., not significant (*P* > 0.05).

The naïve juveniles (16 fish) developed attack side preference during subsequent sessions (Sessions 2–5), which occurred every 2–5 days for about two weeks. Figure 1C and D represent the data during Session 1 and Session 5, respectively. The juveniles during Session 5 successively attacked from the dominant side similarly to wild adults (Supplementary Movie 2). The attack side shifted gradually to the direction of mouth opening (Fig. 2A, Spearman’s rank correlation, ρ = 0.386, *P* < 0.001). In parallel, the number of individuals with significant behavioural laterality (binomial test, *P* < 0.05) increased during the five sessions (3, 5, 10, 12, and 13 of 16 tested fish, respectively). These results suggest that most naïve juveniles acquired behavioural laterality after practice. However, the acquisition of behavioural laterality might be explained merely by an increase in age. To examine this possibility, we tested the first predatory behaviour of naïve adults (64.43 ± 1.25 mm SL, *N* = 6, 9 months old) with no scale-eating experience. As shown in Fig. 2B, the naïve adults exhibited only low preference in attack side during Session 1, similar to naïve juveniles, with no significant difference between them (Wilcoxon rank-sum test: z = −0.808, *P* = 0.419). Furthermore, the behavioural laterality of naïve adults during Session 1 was significantly lower than that of juveniles during Session 5 (Wilcoxon rank-sum test: z = −2.768, *P* = 0.006). To strictly examine the effect of age on behavioural development, we compared behavioural laterality during Session 5 (44.0 ± 0.11 mm SL, *N* = 6, 5 month olds) with that during Session 1 of the same-aged juveniles (46.0 ± 0.09 mm SL, *N* = 6). Again, the bias of attack side during Session 5 was significantly higher than that during Session 1 (Fig. 2C; Wilcoxon signed-rank test: z = −10.50, *P* = 0.016). These results show that the enhancement of behavioural laterality during predation is caused by the scale-eating experience, not by age.

### Lateral difference of predation success in attack direction and kinematics of scale-eating behaviour

**Figure 3:**
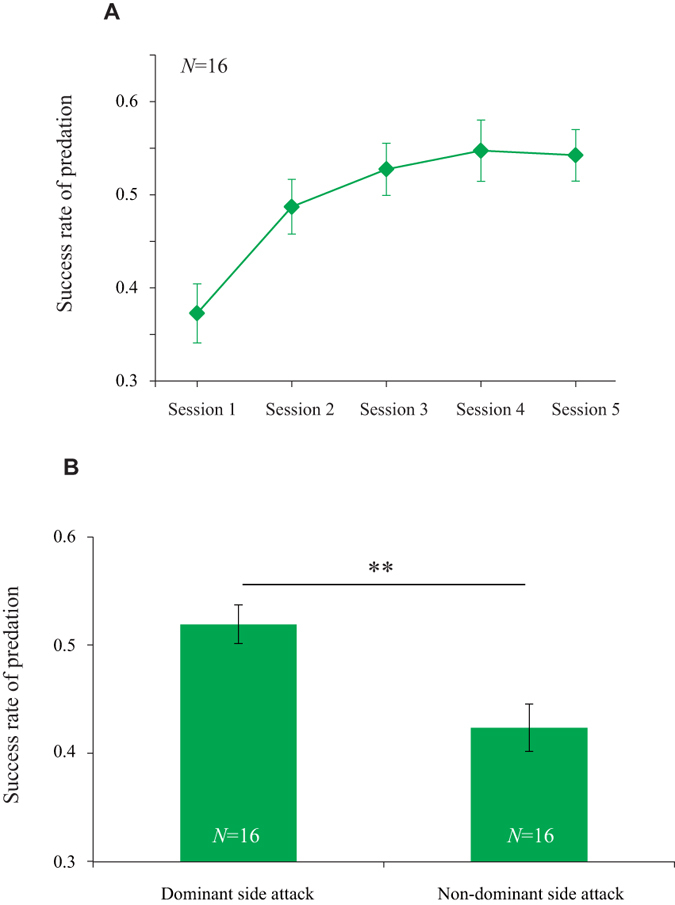
Success rate of predation. (**A**) The temporal change in the success rate of predation from Sessions 1 to 5 (mean ± SE, *N*  = 16 fish). (**B**) The whole success rates of predation from dominant and non-dominant sides (mean ± SE, *N*  = 16 fish). P-values are from GLMM analysis. **P < 0.01.

In parallel with the enhancement of behavioural laterality, the success rate of attacks increased (Fig. 3A, Spearman’s rank correlation, ρ = 0.332, *P* = 0.003), particularly between Sessions 1 and 2. A generalised linear mixed-model (GLMM) analysis was performed to assess the effects of the number of sessions and attack side related to an asymmetric mouth on predation success. The result showed that the success rate from the dominant side of the asymmetric mouth was higher than that of non-dominant side attacks throughout sessions (GLMM analysis, attack side: z = −3.178, *P* = 0.002, session: z = 5.277, *P* < 0.001; Fig. 3B). Thus, the scale-eater is superior in predation on the dominant side during learning.

**Figure 4:**
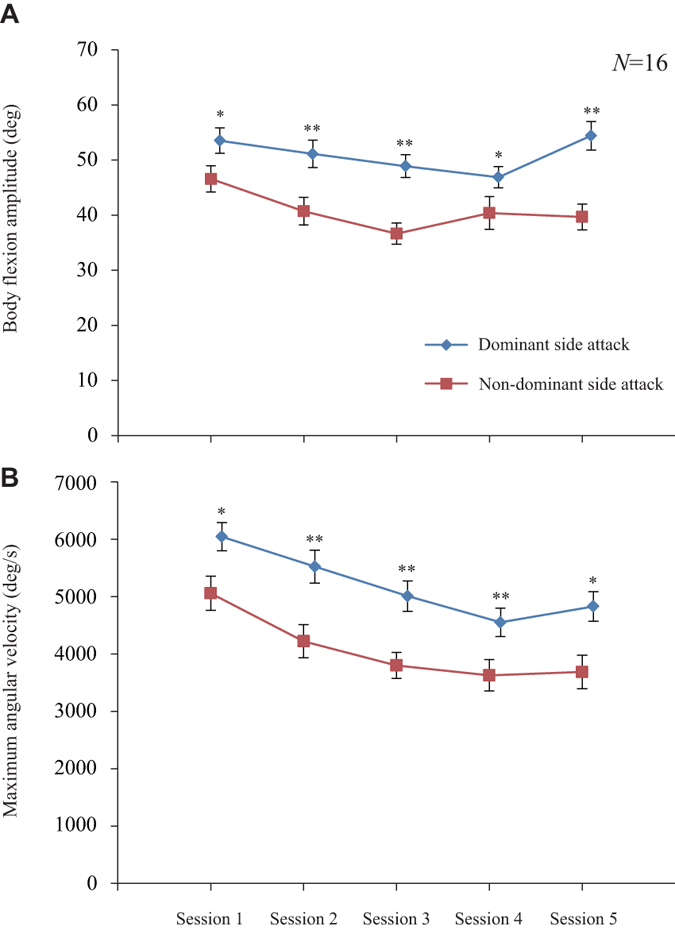
Temporal change in the kinematic difference between a dominant and non-dominant side attack. The change in the amplitude of body flexion (**A**) and maximum angular velocity (**B**) of predation from Sessions 1 to 5 (mean ± SE, *N*  = 16 fish). P-values are from the Wilcoxon rank-sum test between dominant and non-dominant sides. *P < 0.05; **P < 0.01.

Finally, we analysed the kinematics of body flexion of 557 predation events in 16 fish recorded with a high-speed (500 frames/sec) video camera (Supplementary Movies 3 and 4). Rapid and extreme body bending during predation led to predation success. The maximum amplitude and angular velocity of body flexion were attained during the initial bending phase in attack. Notably, the amplitude of body flexion was larger in attacks from the dominant side than in attacks from the non-dominant side (Fig. 4A, Wilcoxon rank-sum test, Session 1: z = −2.318, *P* = 0.020; Session 2: z = −3.261, *P* = 0.001; Session 3: z = −3.617, *P* < 0.001; Session 4: z = −2.312, *P* = 0.021; Session 5: z = −2.931, *P* = 0.003). Similarly, the maximum angular velocity was also higher in attacks from the dominant mouth side than in attacks from the non-dominant side throughout Sessions 1–5 (Fig. 4B, Wilcoxon rank-sum test, Session 1: z = −2.355, *P* = 0.019; Session 2: z = −3.103, *P* = 0.002; Session 3: z = −3.301, *P* < 0.001; Session 4: z = −2.826, *P* = 0.005; Session 5: z = −2.224, *P* = 0.026). Interestingly, the lateral differences in kinetics were already significant during Session 1. Further, the lateral difference in the amplitude of body flexion remained largely unchanged during the sessions (Spearman’s rank correlation, dominant side: ρ = −0.072, *P* = 0.528; non-dominant side: ρ = −0.149, *P* = 0.203), though the angular velocity slightly decreased somewhat as the fish acquired more experience (dominant side: ρ = −0.436, *P* < 0.001; non-dominant side: ρ = −0.336, *P* = 0.003). Therefore, there results indicate that scale-eating fish have a naturally stronger side for attacking prey fish and that they learn to use the dominant side through experience, with some adjustment in dynamics.

## Discussion

Although there are a multitude of reports on behavioural laterality^5^, little is known about how behavioural laterality is acquired during development. In the present study, we demonstrated experimentally that naïve juvenile *P. microlepis*, with no prior scale-eating experience, attacked both sides of prey fish during the first session, and they gradually tended to attack the side that corresponded to the mouth opening direction during subsequent sessions (Figs 1–3). These findings confirm our previous results obtained from stomach content analysis^27^: the stomach contents of early juveniles (22 ≤ SL < 45 mm) collected in the southern end of Lake Tanganyika included scales from both sides, while the foraged scales found in adults (SL > 65 mm) were almost exclusively from one side of the prey fish’s flank. In addition, we demonstrated here that acquisition of the lateralised behaviour did not depend on the age of the juvenile and that naïve adult *P. microlepis* attacked bi-directionally as did naïve juveniles. Thus, the attack side preference of the scale-eater is an acquired trait. Our findings have provided qualitative evidence to support the hypothesis that behavioural laterality is reinforced based on experience during development^17, 33^.

Attack side preference was acquired through several sessions, indicating that *P. microlepis* memorise previous predation results (successes/failures) and learn the better side of prey fish to attack. As shown previously, mice learn to use their dominant paw to take food placed to their front-right or front-left^34, 35^. The learning and memory required to obtain food should have a great effect on an individual’s fitness and facilitate enhanced laterality. Exceptionally, a few juveniles (3/21 fish) exhibited a significant attack side preference even during Session 1: two of them showed continuous improvement until Session 5; the P-values of the binomial test decreased further, and the third one (fish F) exhibited considerably more attacks in Session 1 than in Session 5 (Fig. 1C and D). Thus, a minority of juveniles might learn quickly in only a few trials during Session 1. This is the first report to describe the learning processes of behavioural laterality in fish.

Furthermore, the present study has revealed for the first time a kinematic difference in attack body flexion between the dominant and non-dominant sides of naïve juveniles. The dominant side is identified by the asymmetrical shape of the mouth, which was already apparent in all naïve juveniles tested. The amplitude of body flexion and maximum angular velocity during a dominant side attack always exceeded those of a non-dominant side attack in all sessions (Fig. 4). Lateralised attack with higher motor performance on the dominant side should be advantageous for juveniles to succeed in foraging scales, as shown in adult fish. It was surprising that the dominant side kinetics already exceeded those of the non-dominant side during Session 1. Thus, the lateral difference in kinetics is not explained by learning; instead, it is strongly suggested that the scale-eater intrinsically has a dominant side in terms of motor performance for predation that corresponds to the opening direction of the asymmetrical mouth and that they learn from experience which side is more effective for foraging scales and gradually chose the dominant side by which to attack. Unexpectedly, the maximum angle velocity decreased slightly, which was presumably due to learning the proper attack velocity for successful scale-eating.

Based on these results, we propose the following model for the development of behavioural laterality. First, naïve juvenile *P. microlepis* with no prior scale-eating experience show bidirectional attacks, but they show a lateral difference in the efficiency of foraging scales between attack sides based on a skewed mouth morphology and lateralised kinetics. Second, the scale-eater learns the relationship between attack direction and predation results. Finally, the scale-eater develops a clear preference for dominant-side attacks after acquiring scale-eating experience.

The innate superiority of dominant side attack kinetics may be explained by the lateralised strength of the trunk muscles or functionally lateralised control of the central nervous system (brain and spinal cord). Our previous study^28^ demonstrated that C-shaped flexion during a scale-eating attack is quite similar in kinetics (velocity and amplitude) to the C-shaped bend (C-bend) at the beginning of fast escape behaviour in adult *P. microlepis* and that lefty/righty individuals exhibit equivalent C-bends to both sides. Therefore, muscle activity and basic neural mechanisms in the spinal cord to control the C-bend are bilaterally symmetrical in *P. microlepis*, and it is likely that the asymmetric neural control mechanism is located in the supraspinal brain. Initiation of C-bend during fast escape is triggered by the firing of paired giant hindbrain neurons, called Mauthner cells (M-cells)^36–39^. Thus, it is suggested that the M-cells are involved in controlling the C-bend during scale-eating. The M-cells receive visual input from the retina through the tectum, send axons to the contralateral spinal cord, and connect directly to spinal motor neurons and interneurons that control trunk muscles^40^. Thus, if the M-cells play a key role by triggering attack body bending, one of the bilateral M-cell circuits might be more effective at propagating signals intrinsically and might have already been established before the start of scale-eating.

Taken together, we provide strong evidence for enhanced behavioural laterality during predation based on scale-eating experience. The scale-eating experience had a significant effect on attack side preference, but not body flexion kinetics during predation. The kinetics of body flexion during a dominant side attack naturally outperformed those during a non-dominant side attack. Simple behaviour and identifiable neural circuits to control the scale-eater’s lateralised behaviour may provide valuable material for studying the development of behavioural laterality and its underlying brain mechanisms.

## Methods

### Experimental animals

The adaptive radiation of cichlid fish in Lake Tanganyika has resulted in hundreds of endemic species^41, 42^. Lacustrine cichlid species show surprisingly precise ecological specialisation^43, 44^. *P. microlepis* are widely distributed in Lake Tanganyika and have become specialised at feeding predominantly on scales of other fish^45, 46^. The juvenile and adult scale-eaters used for behavioural experiments were obtained from breeding in our laboratory. The broodstock was collected from Lake Tanganyika (Cameron Bay, Zambia; 8°29′S, 30°27′E) and transported to Japan by a fish dealer. The artificially incubated fish were stored individually in aquaria after hatching and maintained at 27 °C and pH 8.3 in a continuously filtered recirculating system. The aquaria were on a light–dark photoperiod of 12 L:12D. The fish were fed daily with granulated food and small pellets only, so they never encountered prey fish before the first predation experiment (Session 1). The fish were not fed one day before each trial to ensure that they were motivated to eat and would exhibit maximum performance. All experimental procedures were approved by the Toyama University Committee on Animal Research (Approval #A2015MED-47), and the experimental methods were carried out in accordance with the approved guidelines.

### Predation experiment

To clarify the inherent level and development of behavioural laterality, we used juvenile *P. microlepis* scale-eaters at 4 months old, (21 fish) with an SL of 45.98 ± 0.77 mm (mean ± SE), which corresponded to the body size of wild fish that begin foraging for prey fish scales^27, 47^. Before the first predation experiment, the juveniles had no experience with scale eating. A scale-eater and a prey goldfish (*Cyprinus carpio*; 5–6 cm SL) were placed in a 40 × 20-cm tank for the predation experiment. Water was 10 cm deep and maintained at 27 °C. A brown cylinder was set up as a hiding space in the corner of the tank. The experimental tank was illuminated by two halogen lights (HVC-SL; Photron, San Diego, CA, USA) that were oriented diagonally to the tank. The tank was surrounded by a blackout curtain so the subject fish could not see the operator. An experimental arena to observe predatory behaviour was devised as described by Takeuchi *et al*.^28^. Above the arena, a high-speed video camera system (500 frames/s, 1024 × 1024 pixels, NR4-S3; IDT Japan, Tokyo, Japan) was mounted to record the dorsal view of predation. The lateral view of the predatory behaviours was monitored simultaneously with a digital video camera (1920 × 1080 pixels, HDR-XR550V; SONY, Tokyo, Japan) positioned one meter lateral to the tank and recording at 30 frames/s. These images were downloaded to a dedicated computer for data analysis. The predatory behaviours of scale-eaters on the prey goldfish appeared to be the same as those observed in the field^26, 28^.

Prior to the predation experiment, a scale-eater was transferred to the experimental tank to acclimatise for one hour. One prey fish was gently introduced into the opposite corner of the tank, and fish behaviour was then recorded by the cameras for up to one hour. Scale-eaters usually lay hidden in the cylinder at the start of the experiment and displayed predatory behaviour in response to movement of the prey fish. After each observation period, the scale-eater and prey fish were gently captured and returned to their home tanks.

We recorded the side of the prey fish attacked (left/right side, Fig. 1A) and success-or-failure of the predation (hit/miss) for each predatory event. “Hit” or “miss” was identified when the scale-eater’s mouth made contact with the flank of the prey fish or not, respectively^28^. To judge predation success accurately, the scale-eating images taken with the high-speed camera were digitised using behavioural analysis software (Dipp-MotionV2D; Direct Co. Ltd., Tokyo, Japan). For the attack side preference of adults that had accumulated scale-eating experience, we used the predatory experiment data of adults collected from Lake Tanganyika in a previous study^28^.

To investigate the development of lateralised predation, the predation experiment was performed in five sessions (Sessions 1–5) at intervals of 2–5 days (Supplementary Figure 1). Fish were fed daily with only pellets between experiments. Fish of the same age without scale-eating experience were used as control fish (six fish). In addition, 9-month-old adult scale-eaters (six fish, 64.43 ± 1.25 mm SL, mean ± SE) without any scale-eating experience were used as naïve adults.

The degree of behavioural laterality during predation was calculated for each individual as the IAP according to the following equation:

$${\rm{IAP}}={{\rm{A}}}_{{\rm{d}}}/({{\rm{A}}}_{{\rm{d}}}+{{\rm{A}}}_{{\rm{n}}})-0.5,$$

where A_d_ is the number of attacks from the dominant side corresponding to their asymmetric mouth morphology, and A_n_ is the number of attacks from the non-dominant direction of the mouth morphology during predation.

### Assessment of the lateral difference in mouth morphology

*P. microlepis* exhibit remarkable mouth asymmetry^24, 27, 29, 30^, and similar asymmetry is suggested to be shared among numerous fish taxa^48^. A lefty fish was identified by the following three characteristics: the left lower jaw was clearly larger than the right, the left side of the head faced front, and the mouth opened rightward; a righty fish was identified by the opposite characteristics^49^. An individual’s mouth morphology as identified by these traits was always consistent^29^. The nature of this mouth asymmetry has been attributed to lateral differences in the length of the jaw joint^23^. After all behavioural experiments, the scale-eaters were anesthetised in 0.01% tricaine methanesulfonate (MS-222; Sigma-Aldrich, St. Louis, MO, USA), and the mouth and craniofacial morphology were examined visually under a binocular microscope by two researchers (Y.T. and Y.O.). The asymmetry clearly emerged when the fish’s mouth was opened. The mouths of all juveniles observed during the behavioural test opened either to the left side or to the right side: specifically, 12 fish were lefties and nine were righties. Three lefty and three righty naïve adults were used.

### Kinematics of scale-eating behaviour

The scale-eating images taken with the high-speed camera were digitised using kinematic analysis software (Dipp-MotionV2D; Direct Co. Ltd.). In some cases, the movements of the scale-eater were obscured because the images of the two fish overlapped. Only predatory events that were clearly visible from the high-speed camera were used in subsequent analyses. Body flexion angle and angular velocity were measured following Takeuchi *et al*.^28^. Body flexion angles were measured at three points on the midline of the body: the snout, the caudal peduncle, and the centre of mass^50, 51^. The mean centre of the mass of the body of *P. microlepis* was located at a relative distance of 38.3% from the snout^28^. Angular velocity was calculated by dividing the change in the flexion angle observed in five sequential frames by time.

### Statistics

Significant individual preference for attacking a particular prey flank was determined by the binomial test (*P* < 0.05). We also calculated Spearman’s rank correlation coefficient to test whether the degree of behavioural laterality and predatory success temporally changed within the repeated predation experiments. The Wilcoxon signed-rank test was performed to compare the attack side preference between Sessions 5 and 1 of juveniles of the same age. A GLMM analysis was performed to assess the effect of the relationship between the number of sessions and attack side related to mouth asymmetry on the success rate of predation. We designed the GLMM with predation success (hit or miss) as the dependent variable and the following as independent variables: number of sessions (1–5) and attack side related to mouth asymmetry (dominant side or non-dominant side) as the fixed effect and individual as the random effect. The GLMM analysis was performed using the R statistical package (R Statistical Computing, Vienna, Austria). Other statistical analyses were performed using JMP ver.11 (SAS Institute, Cary, NC, USA).

### Data availability

The authors declare that all data supporting the findings of this study are available within the article and its Supplementary Information files.

## Electronic supplementary material

SUPPLEMENTARY INFO

Supplementary Movie 1

Supplementary Movie 2

Supplementary Movie 3

Supplementary Movie 4
