# Supplementary material for: Lateralized scale-eating behaviour of cichlid is acquired by learning to use the naturally stronger side
Source: Sci Rep. 2017 Aug 21;7:8984. doi: 10.1038/s41598-017-09342-7 (PMC5567130; doi:10.1038/s41598-017-09342-7)
Supplement: Supplementary file 1 — SUPPLEMENTARY INFO [file 41598_2017_9342_MOESM1_ESM.pdf]

Supplementary information

Lateralized scale-eating behaviour of cichlid is acquired by learning to use the naturally stronger side

Yuichi Takeuchi[1, 2*] & Yoichi Oda[2]

[1]Department of Anatomy and Neuroscience, Graduate School of Medicine and Pharmaceutical Sciences, University of Toyama, Toyama, Japan

[2]Graduate School of Science, Nagoya University, Aichi, Japan

*Corresponding author

Supplementary Figure 1. Paradigm for the predation experiment. Session 1 for juveniles was conducted about four months after hatching (e.g., “S1” indicates Session 1). The repeated experiments were conducted at intervals of several days. The juveniles spawned on the same day (columns of the same colour) were used for comparison of attack side preference between Sessions 5 and 1 (Fig. 2C). Session 1 for naïve adults was conducted about nine months after hatching. None of the fish had ever encountered prey fish before Session 1.

Supplementary Figure 2. Percentage of left-sided (blue column) and right-sided (red column) attacks for each wild-caught adult (modified from Takeuchi et al. 2012). Each fish attacked more than five times in one hour. Grey columns indicate failed attempts at scale eating. Numbers at the bottoms of the columns indicate the number of attacks by each fish. Asymmetric mouth morphology, lefty (L) or righty (R), is denoted for each fish. *P*-values are from a binomial test. *, $P < 0.05$.

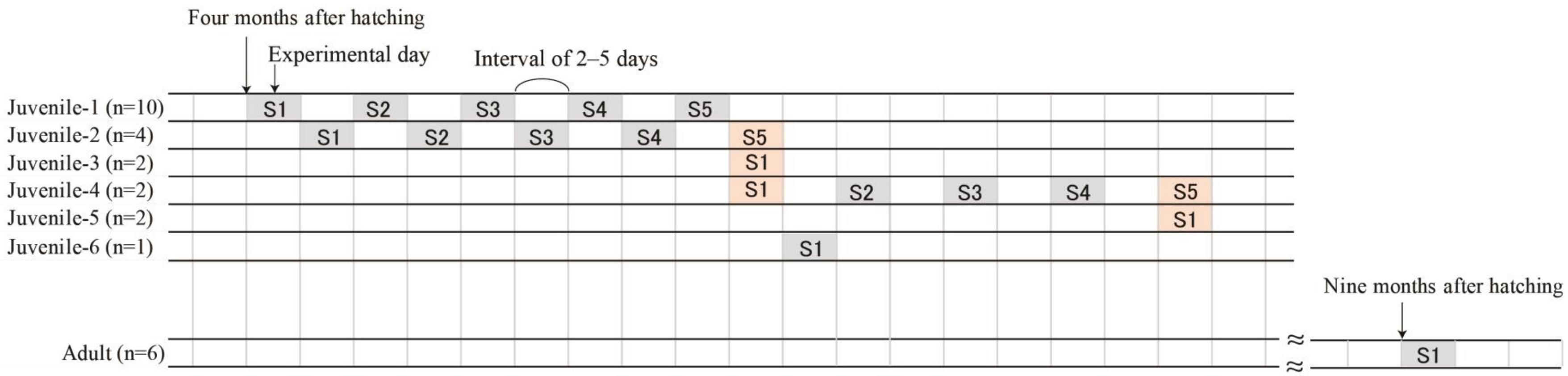

Supplementary Figure 1

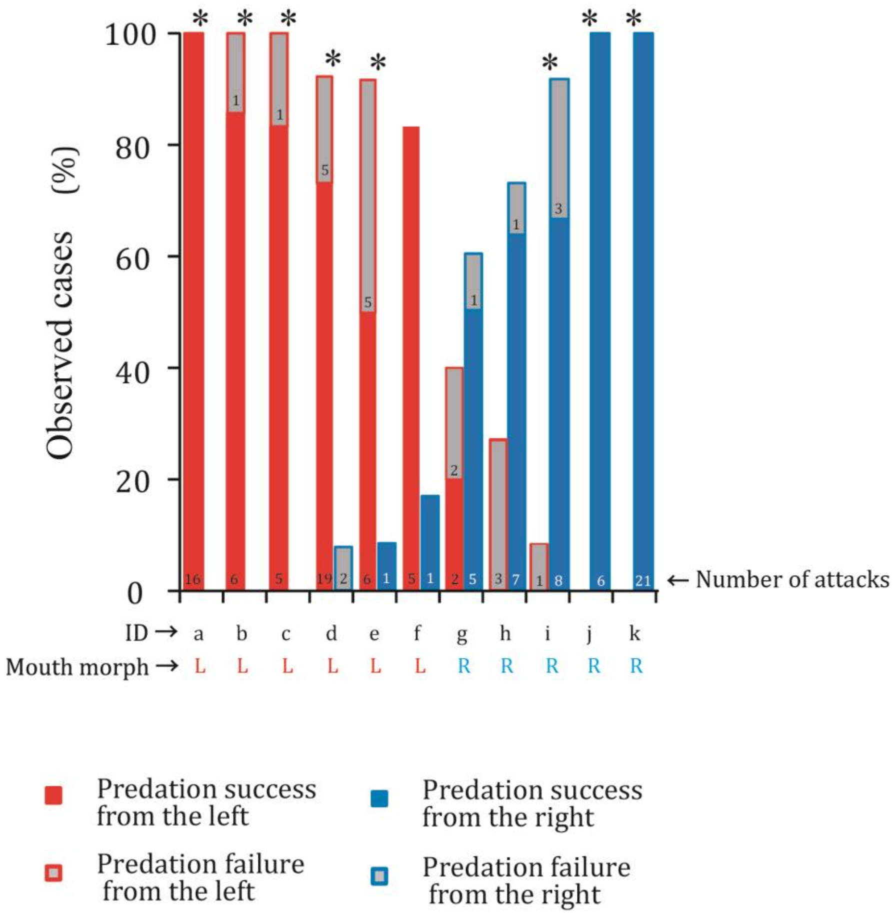

Supplementary Figure 2

Supplementary Movie 1. Bilateral predation behaviour of juveniles during Session 1 (righty). The naïve juvenile attacked from both sides of the prey fish over a short period (right-sided attack and then left-sided attack). The scene is at normal speed. The “GEX” logo presented here was conducted with permission from GEX Corporation Ltd.

Supplementary Movie 2. Lateralised predation behaviour of juvenile during Session 5 (righty). The experienced juvenile consecutively attacked from one side of the prey fish (three consecutive right-sided attacks). The scene is at normal speed. The “GEX” logo presented here was conducted with permission from GEX Corporation Ltd.

Supplementary Movie 3. Predatory behaviour of juvenile (lefty) in Session 1. The dorsal view of predation is in slow playback format (×0.06).

Supplementary Movie 4. Predatory behaviour of juvenile (righty) in Session 1. The experienced scale-eater purposefully approached the right side of the prey. The dorsal view of predation is in slow playback format (×0.06).
